# Supplementary material for: Single-Cell Transcriptional Landscape Reveals the Regulatory Network and Its Heterogeneity of Renal Mitochondrial Damages in Diabetic Kidney Disease
Source: Int J Mol Sci. 2023 Aug 31;24(17):13502. doi: 10.3390/ijms241713502 (PMC10487965; doi:10.3390/ijms241713502)
Supplement: Supplementary file 1 [file ijms-24-13502-s001.zip › ijms-2570725-supplementary.pdf]

Supplemental Figure

Figure S1: Doublets and singlets proportion predicted by DoubletFinder.

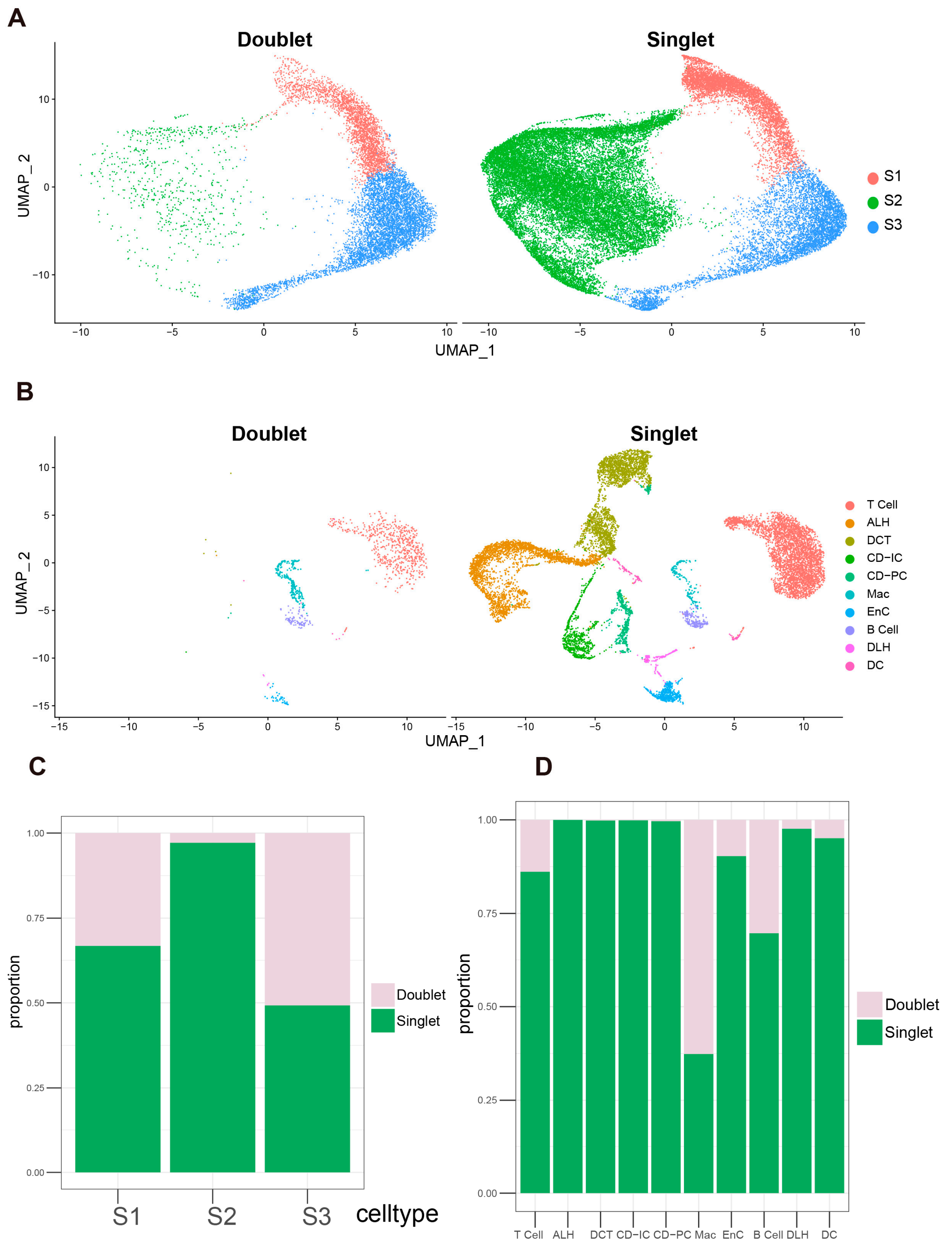

A: UMAP map of S1, S2, S3 clusters showed the projection singlets and doublets in PTCS. B: UMAP map of renal cell types except PTCs showed the projection of singlets and doublets. C-D: The cell proportion stacking diagram showed the proportion of doublets and Singlets to PTCs (C) and renal cell types (D) except PTCs.

Figure S2: Expression of 13 mRNA encoded by mitochondria in 13 renal cells.

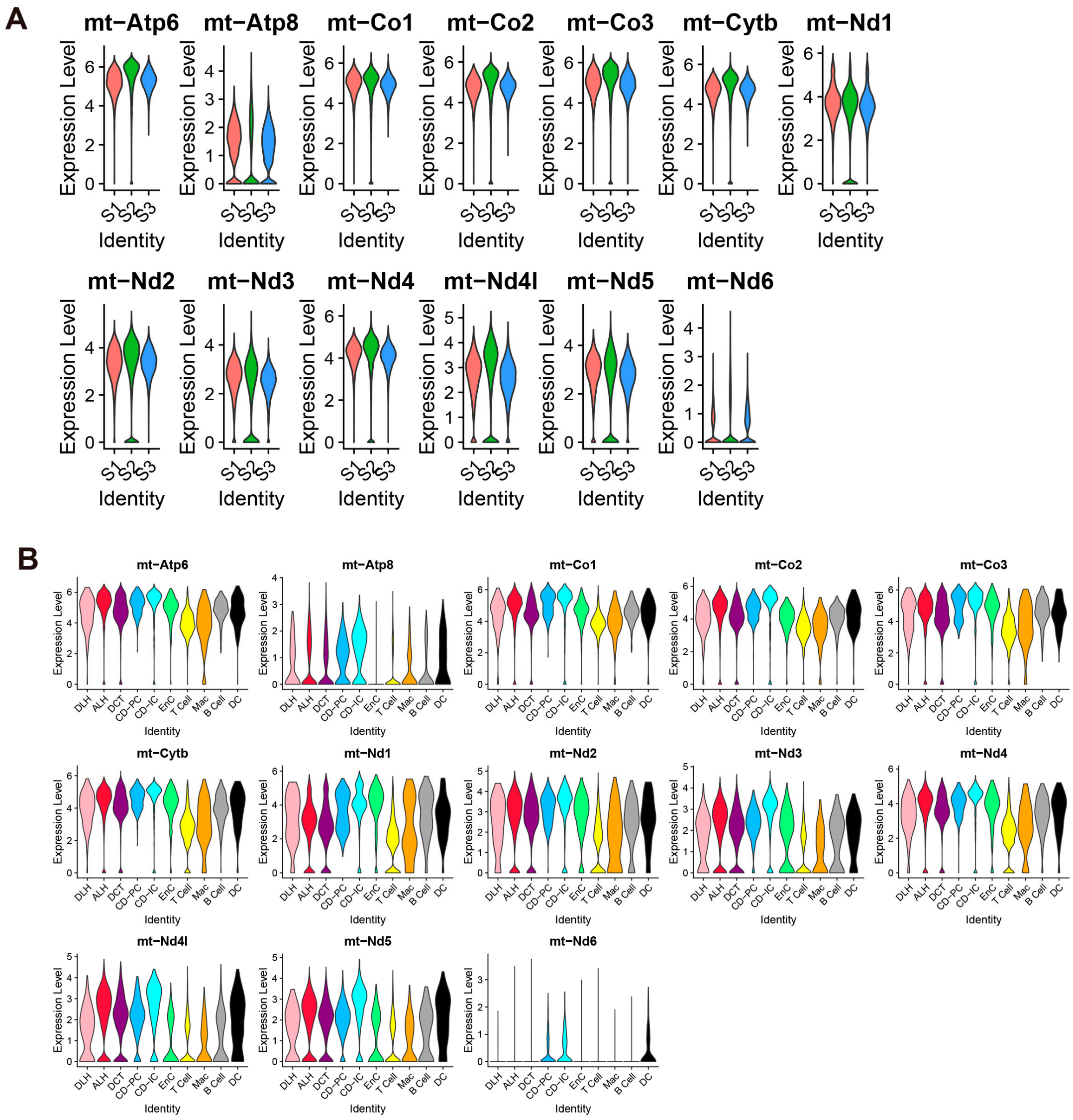

A-B: The expression of genes encoded by mitochondrial genomes in proximal convoluted tubules (A) and other cell types (B) is represented as a violin with no consistent y-coordinate.

Figure S3: Schematic diagram of genes encoding the electron transport chain of mitochondrial oxidative respiration.

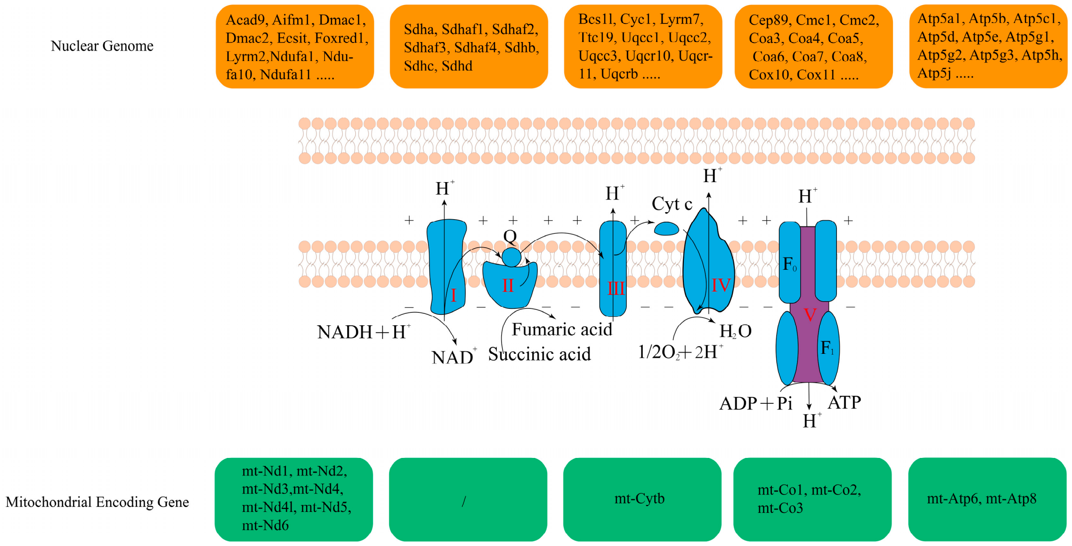

Figure S4: Volcano plot showed differences of the GSVA score of mitochondrial coding gene sets among different cell types between Ctrl group and DKD group.

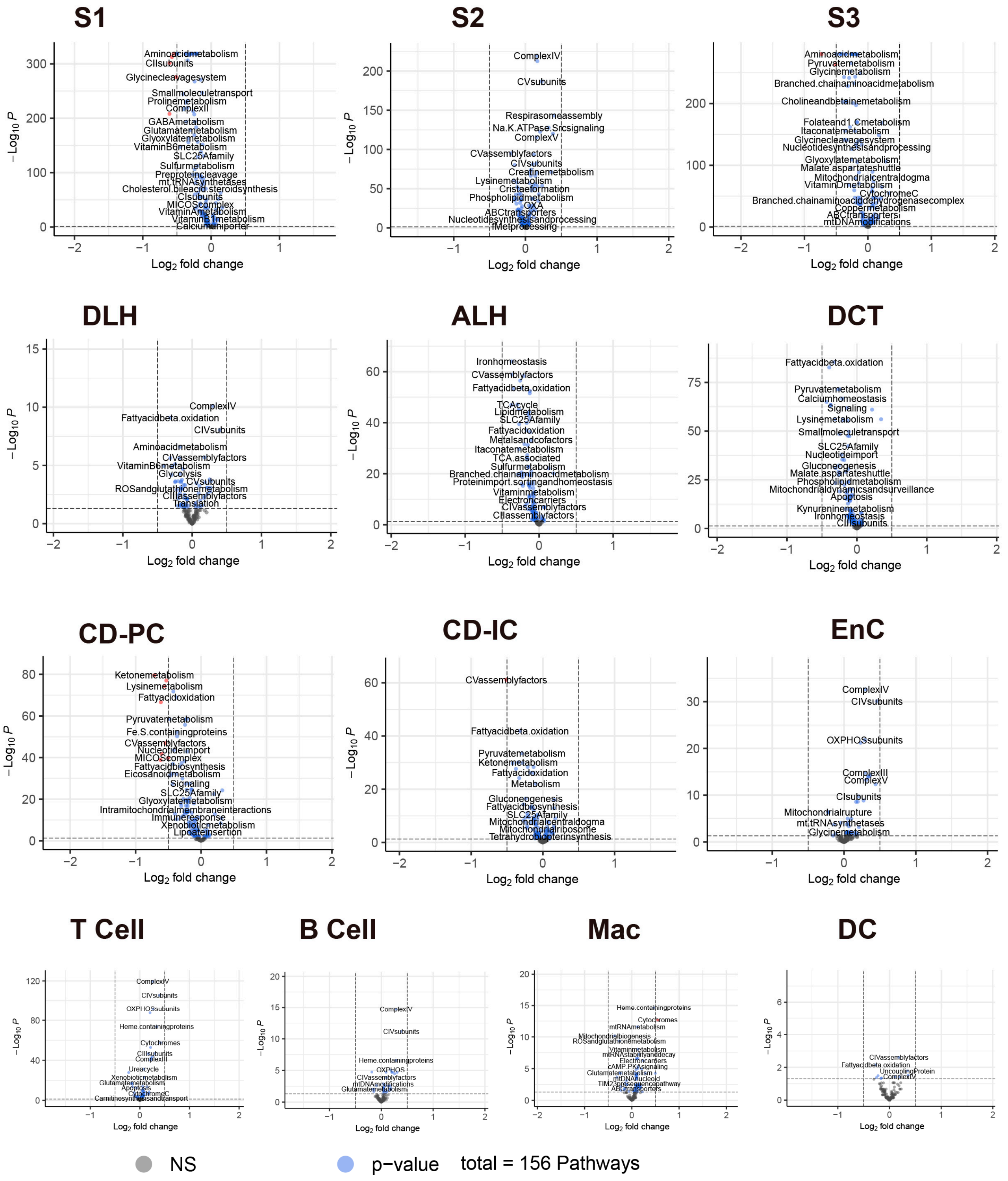

The number of significantly different pathways (P adjustment < 0.05 & |log2FC| > 0.5) was shown in Table 1. The histogram proportional stacking diagram showed the unsupervised clustering results according to GSVA score matrix of nuclear coding gene sets.

Figure S5: The proportion of 13 renal cell types after re-clustering.

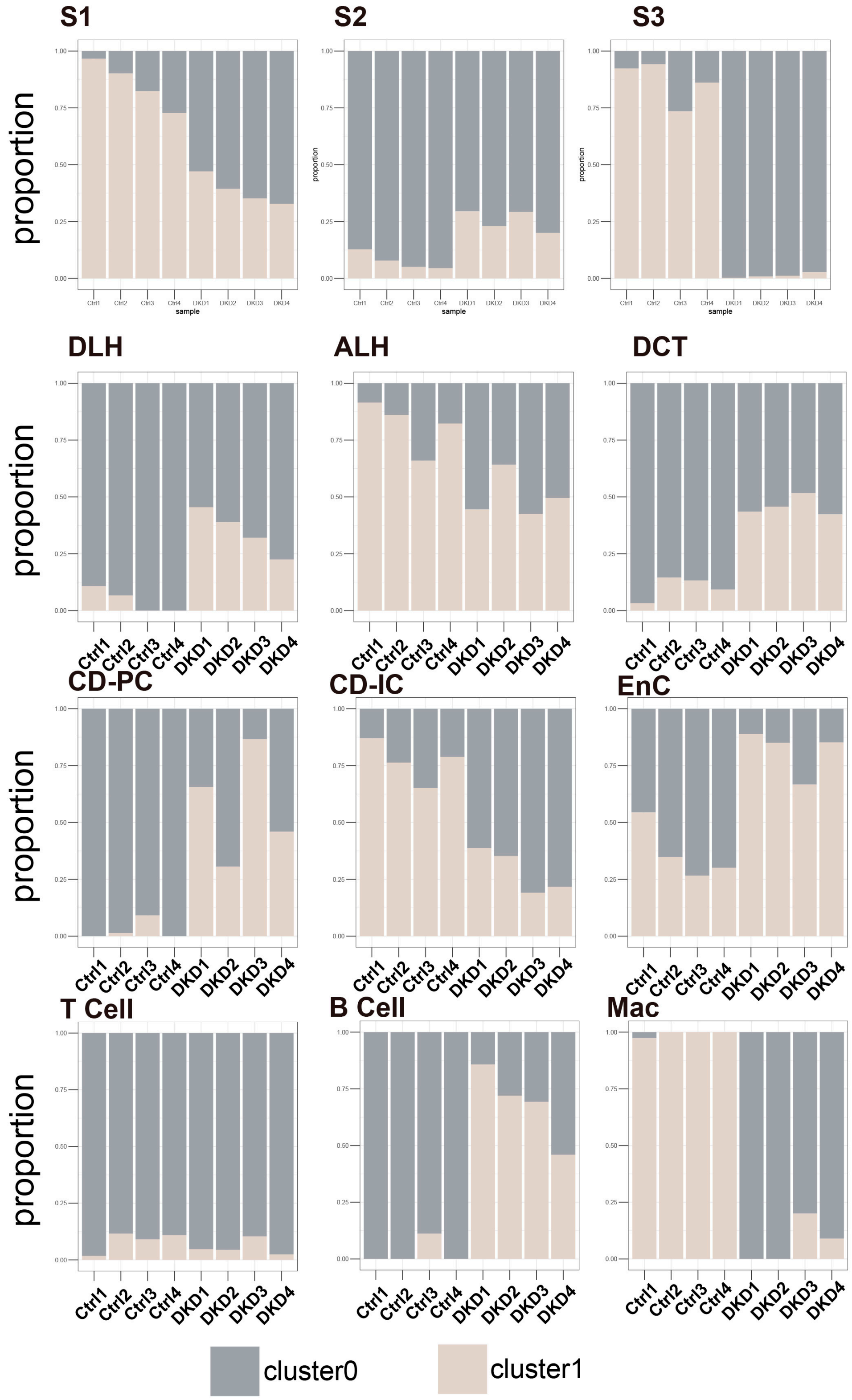

The histogram proportional stacking diagram showed the unsupervised clustering results of GSVA score of nuclear coding gene sets matrix.

Figure S6: SLC superfamily is widely distributed in kidney and differentially expressed between HDC and DDC.

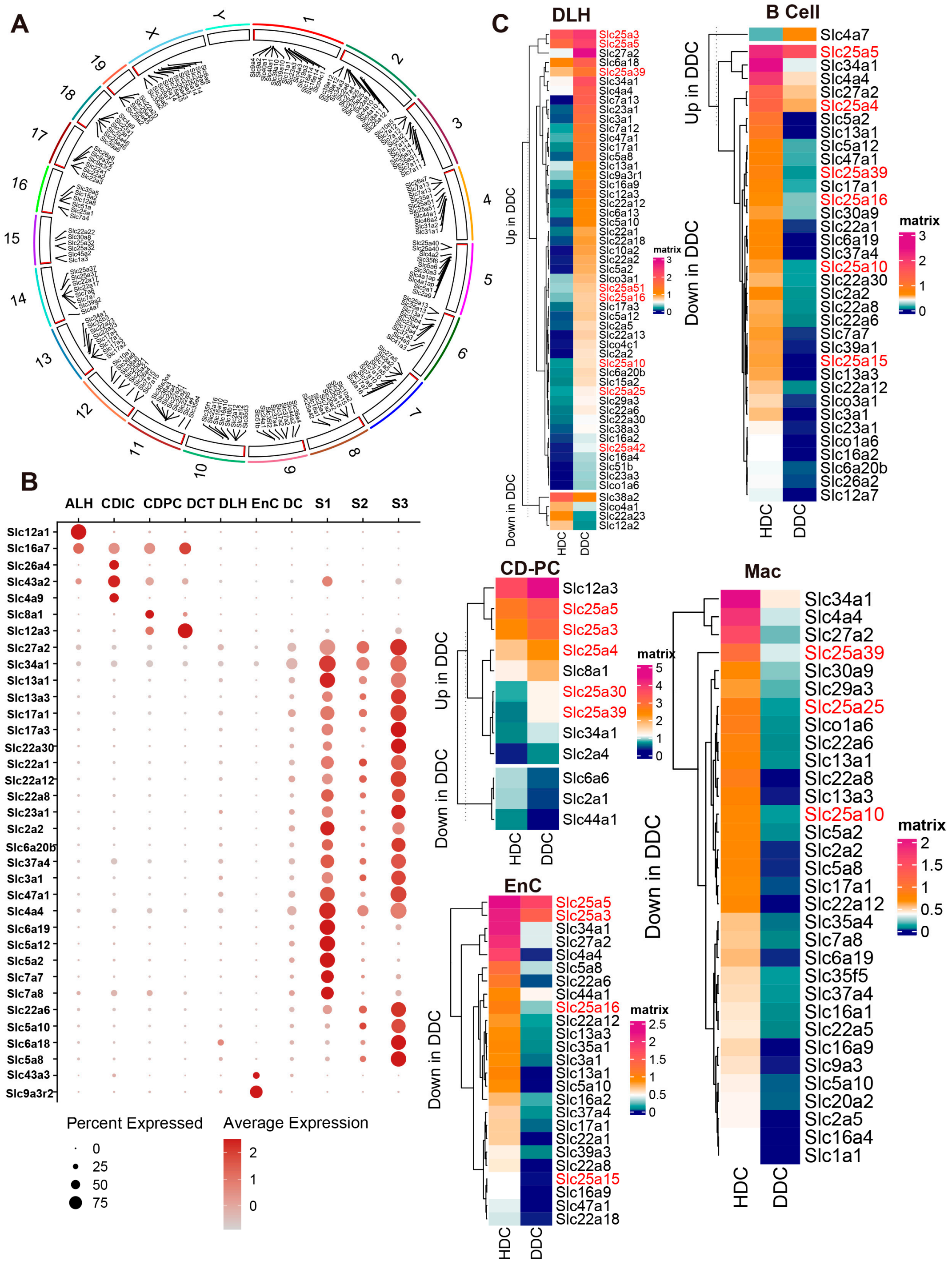

A: Chromosome locations of SLC family. B: Bubble diagrams showed the specific expression of SLC superfamily genes in renal cells. C: The expression levels of differently expressed (P value < 0.05, |Log2(Fold Change)| > 0.5) SLC superfamily in corresponding cells between the HDC and DDC. The genes of SLC25 family were labeled in red.

Figure S7: Cell communication among HDC and DDC of DLH, CD-PC, EnC, B Cell and Mac and other cells.

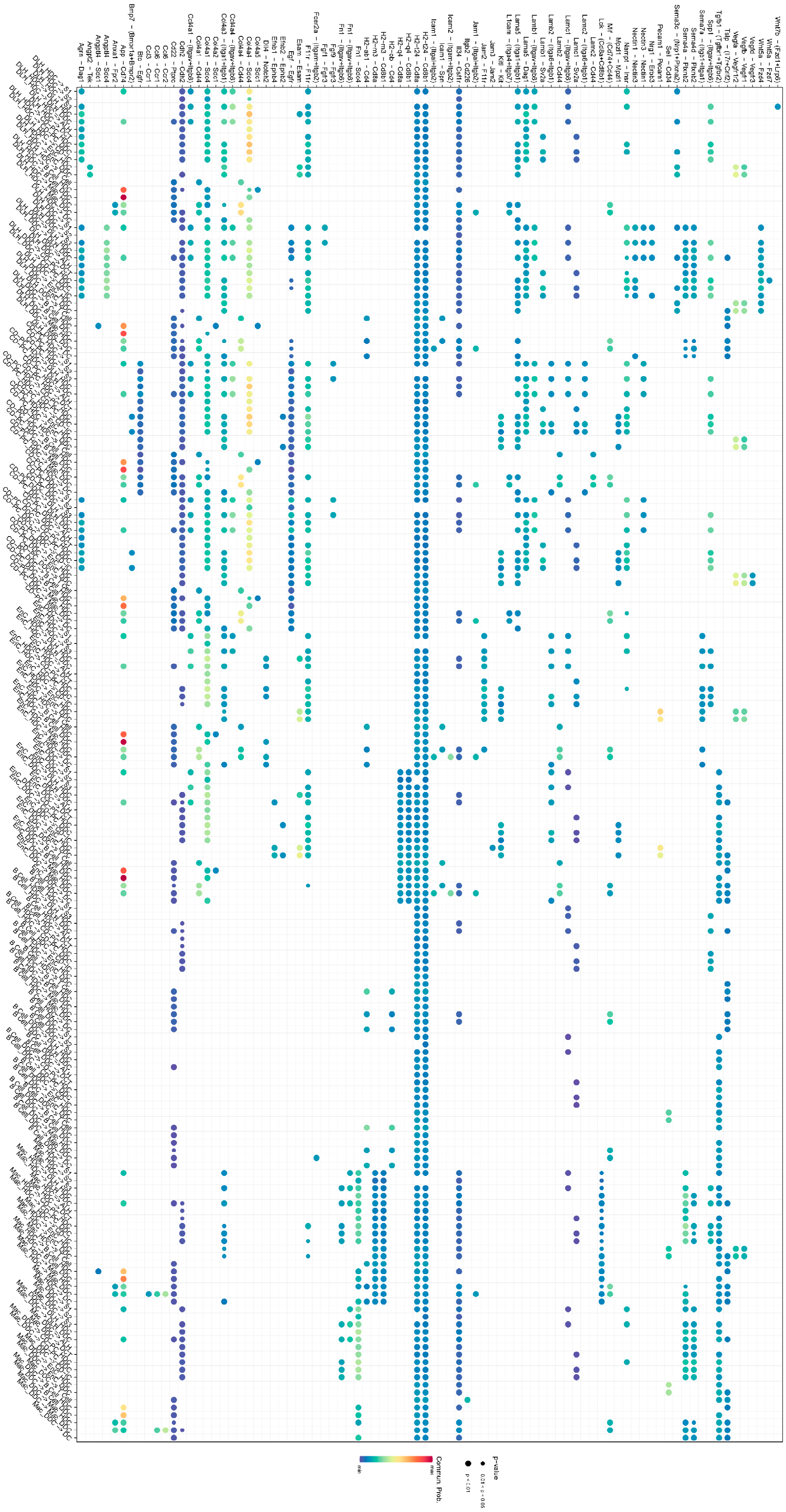

According to calculation of CellChat package, the DKD group contained 46 cellular communication pathways with a total of 90 ligand-receptor pairs.

Figure S8: Continuity matrix of top 50 regulons (if sufficient) calculated by AUCell in SCENIC with significant difference between DDC and HDC.

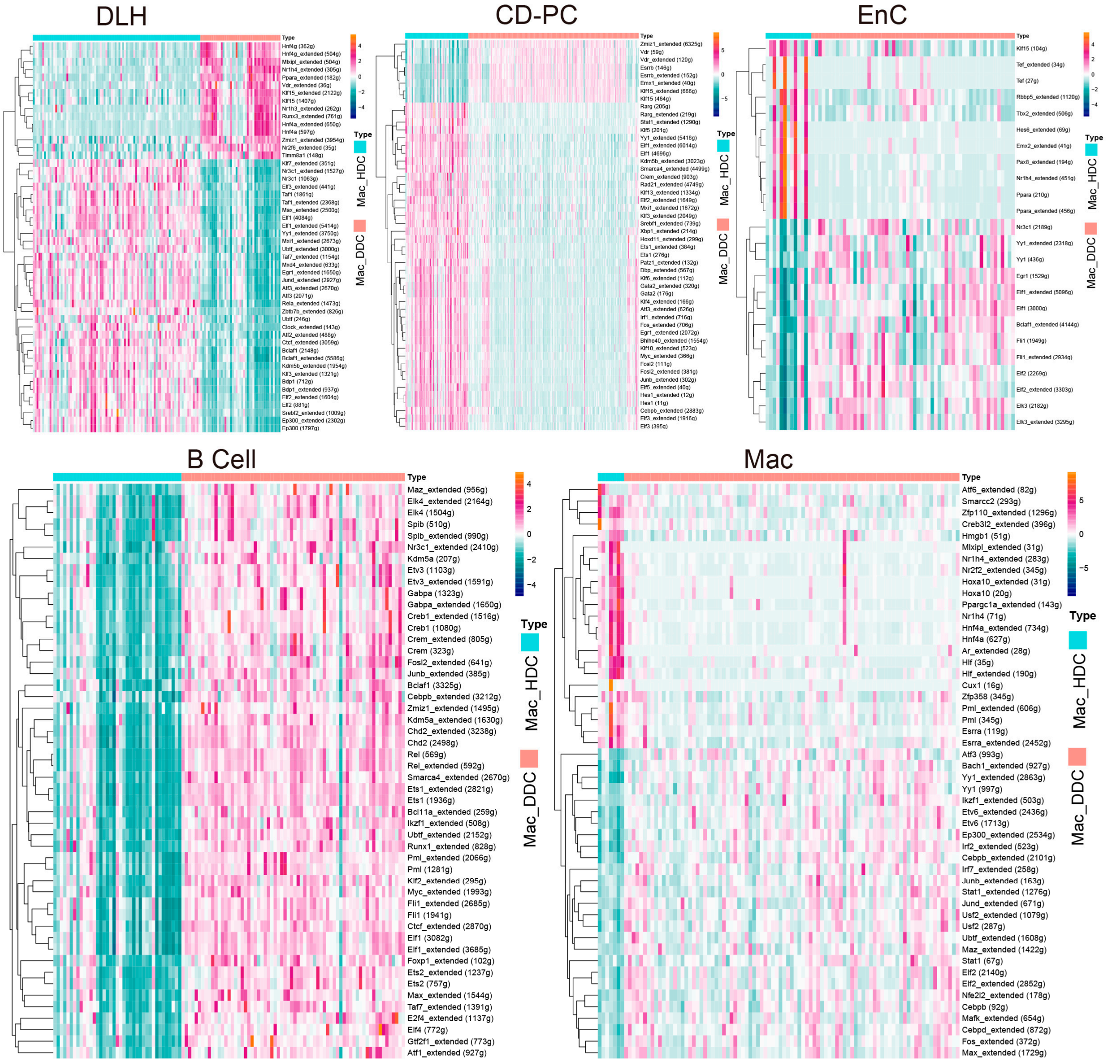

Figure S9: Sankychart shows the complex regulatory network and correspondence among cell receptors, transcription factors and target gene.

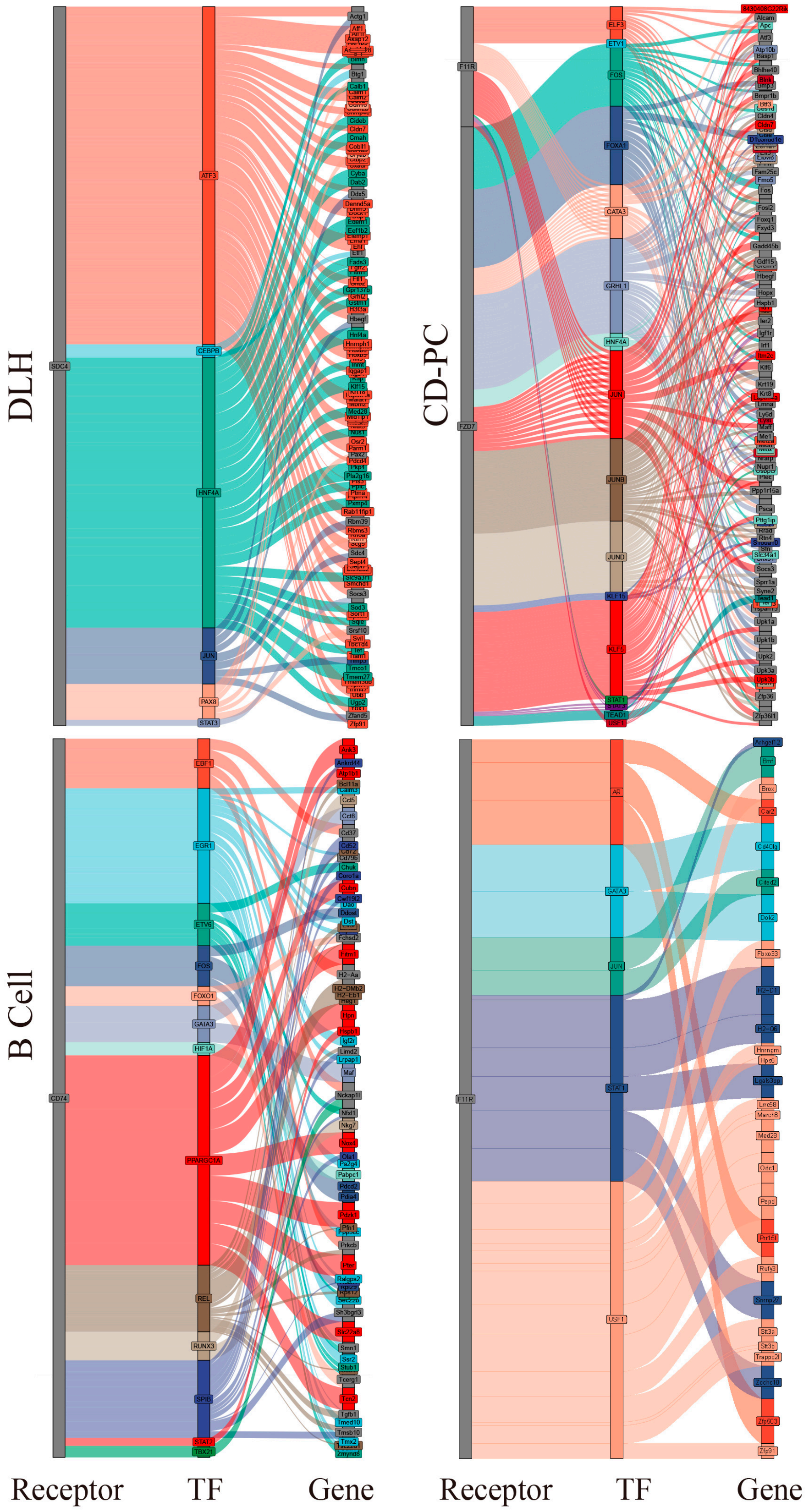

Figure S10 The cascaded regulatory network of receptors, TF and TG resulted in the regulation of specific KEGG pathways and cell state of HDC and DDC.

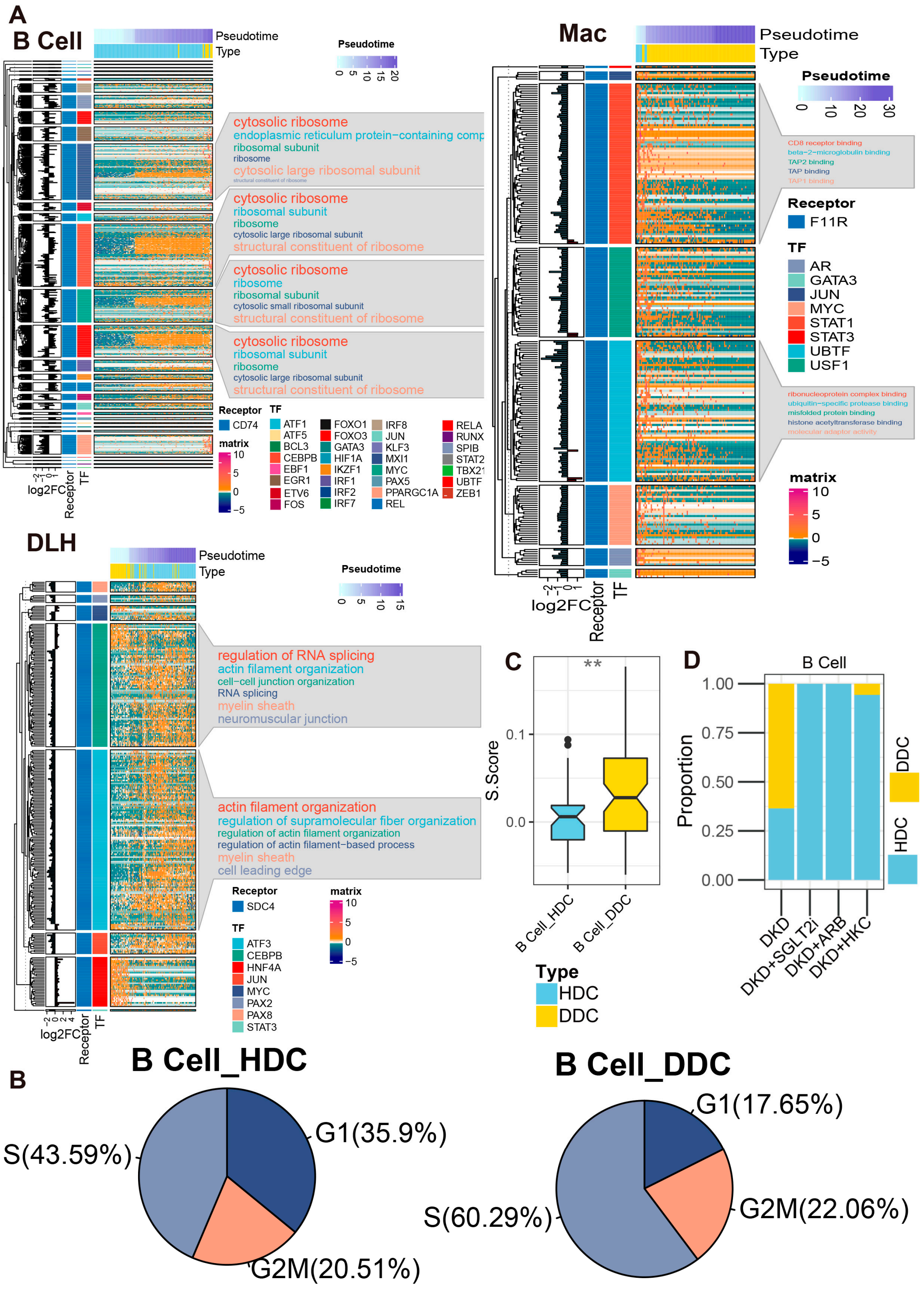

A: The complex heat map showed the distribution of HDC and DDC of DLH, B cell, Mac as well as the expression of ESDEG with the progression of pseudotime. The four columns of left annotation from left to right are kmean clustering tree, the fold change of ESDEG to HDC in DDC, receptor and TF respectively. More than 50 genes belonging to the same transcription

factor will be enabled for KEGG enrichment analysis and shown in the word cloud annotation on the right. B: Pie charts of cell cycle distribution of two subpopulations of B cell in DKD group. The S-phase cell proportion of B cell_DDC was significantly higher than what of B cell_HDC. C: The S-phase score calculated by cell cycle signatures was significantly higher in B cell _DDC than in B cell_HDC. (**, $P < 0.01$, T test). D: The distribution of HDC and DDC in B Cell in ARB, SGLT2i, and HKC groups was predicted by singleR.
